# Improved sports image classification using deep neural network and novel tuna swarm optimization

**DOI:** 10.1038/s41598-024-64826-7

**Published:** 2024-06-19

**Authors:** Zetian Zhou, Heqing Zhang, Mehdi Effatparvar

**Affiliations:** 1 School of Physical Education and Sports Science, South China Normal University, Guangzhou, 510631 Guangdong China; 2 Management School of Guangzhou University, Guangzhou, 510006 Guangdong China; 3 Department of Computer, Islamic Azad University, Ardabil Branch, Ardabil, Iran; 4 College of Technical Engineering, The Islamic University, Najaf, Iraq

**Keywords:** Sports image classification, Deep neural network, Novel tuna swarm optimization, Hyperparameter optimization, Performance evaluation, Energy science and technology, Engineering

## Abstract

Sports image classification is a complex undertaking that necessitates the utilization of precise and robust techniques to differentiate between various sports activities. This study introduces a novel approach that combines the deep neural network (DNN) with a modified metaheuristic algorithm known as novel tuna swarm optimization (NTSO) for the purpose of sports image classification. The DNN is a potent technique capable of extracting high-level features from raw images, while the NTSO algorithm optimizes the hyperparameters of the DNN, including the number of layers, neurons, and activation functions. Through the application of NTSO to the DNN, a finely-tuned network is developed, exhibiting exceptional performance in sports image classification. Rigorous experiments have been conducted on an extensive dataset of sports images, and the obtained results have been compared against other state-of-the-art methods, including Attention-based graph convolution-guided third-order hourglass network (AGTH-Net), particle swarm optimization algorithm (PSO), YOLOv5 backbone and SPD-Conv, and Depth Learning (DL). According to a fivefold cross-validation technique, the DNN/NTSO model provided remarkable precision, recall, and F1-score results: 97.665 ± 0.352%, 95.400 ± 0.374%, and 0.8787 ± 0.0031, respectively. Detailed comparisons reveal the DNN/NTSO model's superiority toward various performance metrics, solidifying its standing as a top choice for sports image classification tasks. Based on the practical dataset, the DNN/NTSO model has been successfully evaluated in real-world scenarios, showcasing its resilience and flexibility in various sports categories. Its capacity to uphold precision in dynamic settings, where elements like lighting, backdrop, and motion blur are prominent, highlights its utility. The model's scalability and efficiency in analyzing images from live sports competitions additionally validate its suitability for integration into real-time sports analytics and media platforms. This research not only confirms the theoretical superiority of the DNN/NTSO model but also its pragmatic effectiveness in a wide array of demanding sports image classification assignments.

## Introduction

The precise categorization of sports images is a complex task that requires advanced methodologies for efficient feature extraction and accurate classification. The proliferation of sports-related content across various media platforms has highlighted the need for effective techniques to categorize sports images^1^. However, the inherent intricacies of sports imagery, such as dynamic movements, diverse backgrounds, and variable lighting conditions, pose significant challenges for conventional image classification techniques.

The complexities of sports images often make it difficult to accurately capture and interpret the key distinguishing features that define various sports activities. This challenge is further compounded by the need for precise identification and classification of subtle differences in actions, gestures, and playing environments within the context of different sports disciplines^2^. Therefore, the development of sophisticated approaches capable of extracting intricate features and discerning nuanced patterns from sports images is crucial in the realm of modern image processing and computer vision^3^. These advancements aim to facilitate more precise and effective categorization of sports imagery, catering to the increasing demands of sports enthusiasts, media professionals, and related industries for high-quality and accurately classified sports-related content.

It is essential for modern computer vision research to have advanced techniques that can extract significant features and classify sports images with precision, making it a crucial aspect to consider^4^. Deep learning methods have been suggested as a solution to this obstacle, as they enable the automatic acquisition of advanced features from images through the utilization of multiple layers of nonlinear transformations^5^. The effectiveness of deep learning methods extends beyond image classification, encompassing various computer vision tasks including image segmentation, image generation, and image captioning. There are different types of these technique that have been used for sports image classification.

For instance, Gao et al.^6^ directed their attention towards addressing the drawbacks associated with manual techniques for classifying sports videos. Recognizing the importance of automated classification in a range of applications, such as switched TV, video on demand, and smart TV, the researchers introduced a novel classification model called AGTH-Net. This model incorporated an attention mechanism within the graph convolution model to enhance the accuracy of classification. By allocating weights to neighboring nodes effectively and minimizing the influence of error nodes, the attention mechanism improved the overall performance of the model. Additionally, the researchers utilized the third-order hourglass network structure to extract and combine multiscale characteristics specific to sports video images. The integration of residual-intensive modules within the network further enhances its expressive capacity and optimizes feature extraction. However, it is important to note that the proposed AGTH-Net model may face challenges related to computational complexity and the requirement for extensive computational resources. These factors could potentially limit its real-time applicability in large-scale video classification scenarios.

Lei et al.^7^ addressed a practical application of sports practice through the use of image detection technology, broadcast data detection, and video analysis. The study focused on the utilization of video cloth for motion picture detection, including grayscale processing, detection, target identification, light detection era observation, and project grasping. The primary objective of the research was to address the diverse requirements of sports image detection, particularly in facilitating athlete popularity and behavior recognition in sports programs. The researchers established a comprehensive platform for inspection, emphasizing its practicality and potential to serve as a theoretical foundation for future investigations. Additionally, the study introduced a hierarchical set of rules, incorporating the Particle Swarm Optimization (PSO) algorithm for image annotation and daily evaluation. While the results demonstrated the effectiveness of the algorithm, potential limitations of this work may include the need for further refinement and validation of the proposed methodology in diverse sports contexts. It is important to consider the dynamic and multifaceted nature of sports activities when applying this approach. Furthermore, the practical applicability of the approach in real-world settings and its adaptability to various sports scenarios could warrant additional scrutiny and experimentation to ensure its robustness and reliability.

Liu et al.^8^ developed a dataset of football player numbers and identify football players based on their jersey numbers. The dataset construction involved player detection and number region detection. To identify players, the study utilized a section of the YOLOv5 model's backbone network as the feature extraction module, which improved recognition performance by incorporating the SPD-Conv module, particularly beneficial for small-sized targets and low-resolution conditions. The model’s performance was evaluated through a series of experiments, resulting in a recognition accuracy of 92.75%. However, potential limitations of this work may include the need for further validation of the proposed model in diverse football match scenarios, player poses jersey designs, and considering varying lighting conditions, to ensure its robustness and applicability in real-world football settings. Additionally, the generalizability of the model to other sports and its scalability to accommodate larger datasets with increased complexity may require additional investigation and refinement to enhance its versatility and effectiveness in broader sports-related applications.

Yang et al.^9^ proposed a new approach to identifying and positioning table tennis using a convolutional neural network (CNN). The method effectively overcame the limitations of existing techniques that rely on color and contour features, which lack adaptability in diverse environments. The study explored various learning methodologies and techniques for table tennis detection, positioning, and trajectory prediction. Additionally, the researchers proposed a deep learning framework specifically designed for recognizing the intricate motion patterns of spinning table tennis balls. They investigated the strategies and mechanisms for trajectory prediction, precise positioning, and intelligent automated processing of dynamic images, while also meticulously constructing and validating their own datasets. However, potential limitations of this work might include challenges related to the scalability and generalization of the proposed CNN model to a broader range of table tennis environments, as well as the need for further validation in real-world settings to assess its practical applicability and robustness in diverse playing conditions.

Ergeneci et al.^10^ shown that deep neural networks (DNN) are highly accurate in motion classification. However, their reliance on large datasets presents challenges when dealing with limited training data. Due to privacy concerns in professional athlete training, traditional DNN architectures are not suitable for real-life sports applications. To overcome this, the study utilized few-shot learning (FSL) techniques, which uses knowledge from various tasks to classify unseen tasks with minimal data samples. By using a siamese network and triplet loss, the FSL approach achieved superior performance, with median F1-scores of 72.01%, 76%, and 79% for 1, 5, and 10 shot datasets, respectively, even for previously unseen tasks. In comparison, the DNN with transfer learning (TF) had lower performance, with F1-scores of 49.27%, 51.58%, and 67.66% for the corresponding datasets. However, it is important to consider potential limitations of this work, such as further investigations into the scalability and generalizability of the FSL approach to a broader range of sports-related tasks. This is necessary due to the diverse nature of athlete movements and the need for continuous adaptation to new exercise regimes. Additionally, the practical implementation and real-time applicability of the FSL technique in dynamic sports scenarios may require additional optimization to ensure its reliability and effectiveness in various competitive sports environments.

Despite the significant progress made in image classification, categorizing sports images accurately remains a difficult task. The current methodologies often fail to capture the complex features and dynamic elements present in sports images, resulting in suboptimal classification outcomes. The intricate nature of sports images, such as diverse lighting conditions, varying backgrounds, and dynamic motion patterns, requires the development of innovative approaches that can effectively handle these challenges and produce superior classification results. The research gap identified highlights the urgent need for novel methodologies that can bridge the existing gap in sports image classification and enable more precise and efficient categorization of sports-related content.

Deep neural networks (DNNs) are extensively utilized and extensively studied models among the deep learning methods, owing to their remarkable representation and generalization capabilities. Various optimization algorithms, including gradient descent, stochastic gradient descent, and Adam, can be employed to train DNNs. Nevertheless, these optimization algorithms may encounter certain limitations, such as slow convergence, sensitivity to initial conditions, and susceptibility to local optima.

To overcome these limitations, metaheuristic algorithms have been employed to optimize the hyperparameters of DNNs, such as the number of hidden layers, the number of neurons, the learning rate, and the activation function. Metaheuristic algorithms draw inspiration from natural phenomena or biological behaviors, enabling them to efficiently and effectively explore the search space without relying on gradient information or problem-specific knowledge. Genetic algorithm, particle swarm optimization, ant colony optimization, and simulated annealing are some examples of metaheuristic algorithms. However, most of these metaheuristic algorithms are based on simple and linear models, which may not be capable of handling the complex and nonlinear nature of DNNs.

**Figure 1:**
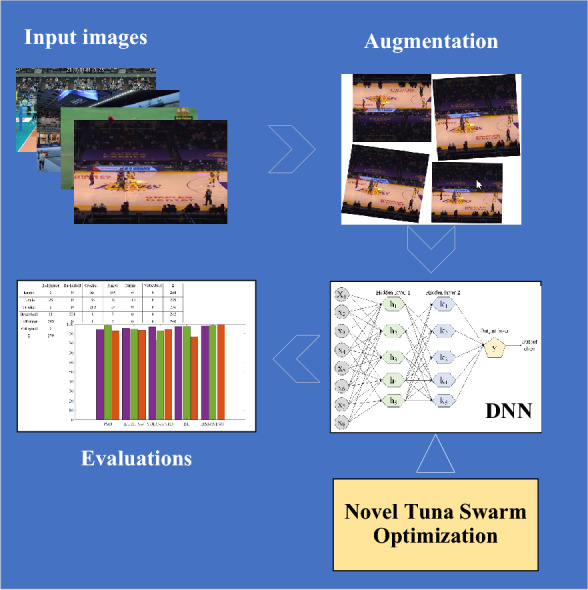
The graphical abstract of the proposed methodology in the present paper.

Nevertheless, sports image classification encounters multiple obstacles arising from intricate movements, diverse camera angles, fluctuating environmental conditions, and overlapping scenes. Conventional machine learning techniques struggle with limited generalization capabilities, whereas deep learning approaches usually demand extensive labeled data and substantial computational resources. Motivated by the increasing demand for precise techniques in classifying sports images, this research, this research introduces an innovative deep learning model named DNN/NTSO for sports image classification, which merges the advantages of a deep neural network (DNN) with a tuna swarm optimization (NTSO) algorithm. Figure 1 shows the graphical abstract of the proposed methodology in the present paper.

The main scope of this paper is highlighted in the following:

- Creating a strong and precise deep learning model for sports image classification that can handle challenges such as complex movements, various camera angles, and changing environmental conditions.
- Developing a tuna swarm optimization (NTSO) algorithm to fine-tune the parameters and weights of the DNN, improving its ability to differentiate between different sports categories.
- Conducting comprehensive experiments and statistical analyses to verify the effectiveness of the proposed DNN/NTSO model and comparing it with other cutting-edge models.
- Exploring the impact of different training setups and hyperparameters on the model's performance and providing recommendations for professionals working on similar projects.

## Database

### Dataset description

**Figure 2:**
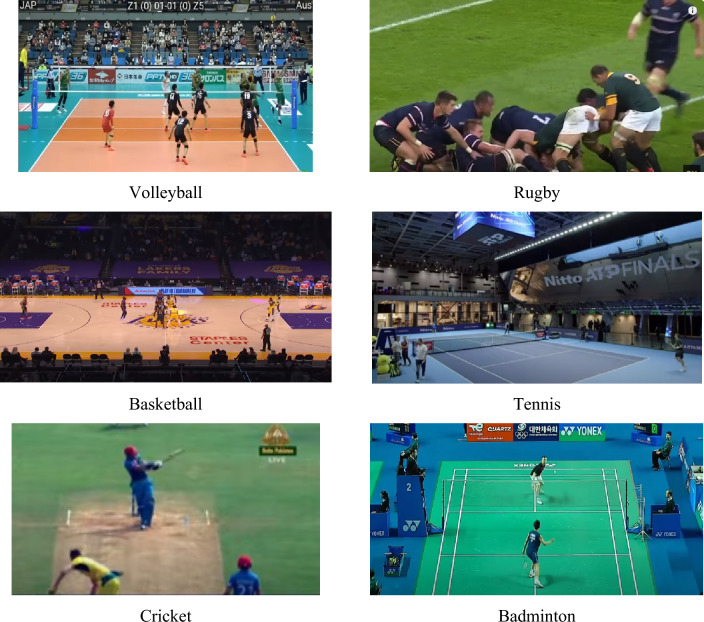
Sample frames depicting diverse image categories.

The high-resolution images in this dataset measure 240 × 320 pixels and have been extracted from a collection of sports videos sourced from various channels, including renowned platforms like [ESPN], [Olympic Channel], and [FIBA]. The dataset comprises lots of frames, each assigned one of six distinct labels representing different sports activities, with a balanced distribution of images across each label. This dataset is primarily designed for classification applications in the sports domain, enabling tasks such as identifying the sport type, recognizing players and teams, and conducting performance and tactical analyses. Despite the presence of low-quality images in some cases, diverse lighting conditions, fluctuating clarity, and intricate backgrounds, this dataset presents an opportunity to evaluate the effectiveness of various image processing and classification methodologies. Figure 2 illustrates sample frames depicting diverse image categories are exhibited.

The process of dataset creation entailed the procurement of videos from the videos uploaded in YouTube that encompassed a wide array of sport categories. The chosen sports, namely Basketball^11^, Rugby^12^, Badminton^13^, Volleyball^14^, Cricket^15^, and Tennis^16^, were carefully selected to ensure a comprehensive representation of various physical activities and game dynamics. In order to construct the dataset, frames were extracted from each video. These frames can be described as individual snapshots captured at specific moments in time during the playback of the video. As a result of this extraction process, a significant number of frames were made accessible for investigation.

It is crucial to partition the dataset into separate test and training subsets, as per standard practices in classification tasks, to confirm precise evaluation of classification models. The entire dataset comprises of 7083 frames. The training set constitutes 80% of the frames, while the test set is composed of the remaining 20%. This division ensures that the models are trained on a substantial amount of data and evaluated on unseen samples that mimic real-world scenarios.

### Data augmentation

This study employs data augmentation to enrich the quantity of the database images by introducing diverse abnormalities. The images experienced alteration over a range of augmentation methods, as well as random X and Y-reflection, scale, rotation, translation, and shear. These procedures were chosen to increase the reliability and diversity of the dataset by introducing variations in the size, orientation, and shape of the images. The imagery underwent random rotation within an angular range from − 90 to 90 degrees.

This allowed the model to acquire knowledge from the images exhibiting diverse rotation angles, hence enhancing its capacity to identify and categorize. In addition, the pictures underwent random shearing along the X and Y axes, with a factor range of − 0.02 to 0.02, in order to replicate abnormalities that may potentially arise from bad capturing or motion. This update has improved the model's capacity to efficiently handle such variances.

In order to enhance the dataset, supplementary photos were included by using random vertical or horizontal reflection to provide alternative views^17^. The use of this augmentation strategy resulted in an improvement in the model's capacity to adapt and generalize across different spatial orientations. Furthermore, the pictures underwent random translations along the X and Y axes, resulting in shifts in both horizontal and vertical orientations.

**Figure 3:**
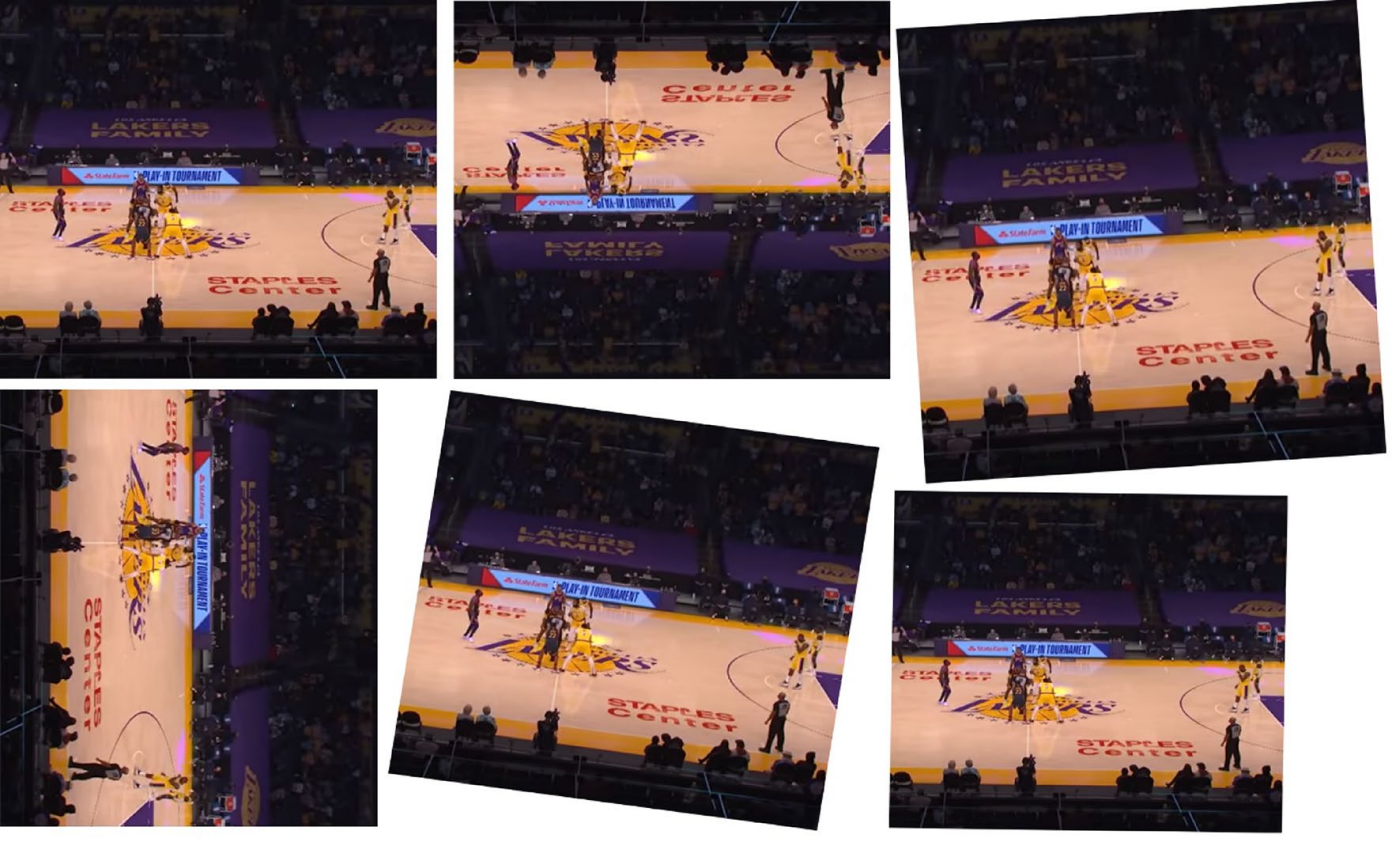
Various sports images sourced from different YouTube databases, serving the purpose of classification.

In addition, the photos underwent random scaling along the X and Y axes, resulting in alterations to their dimensions but preserving the original aspect ratio. Figure 3 showcases various sports images sourced from different YouTube databases, serving the purpose of classification.

Each sub-image in Fig. 3 depicts a unique augmented sport image, as evident. For every image, multiple variations are showcased, which are produced using data augmentation techniques. By analyzing these sub-figures, we can visually comprehend the diversity and variability that is incorporated into the dataset through the application of data augmentation. Table 1 presents an extensive statistical examination of the dataset, furnishing intricate insights into its composition and distribution.

**Table 1:** Extensive statistical examination of the dataset, furnishing intricate insights into its composition and distribution.

Category	Training images	Test images	Total images
Rugby	1220	976	244
Tennis	1145	916	229
Crocket	1180	944	236
Badminton	1200	960	240
Basketball	1210	968	242
Volleyball	1128	902	226
Total	7083	5666	1417

As can be observed from Table 1, there is a balanced distribution, indicating that each category contains a comparable number of images. Moreover, both the training and test sets maintain an equal proportion of images across categories. This ensures that the dataset accurately represents real-world scenarios and prevents any bias towards specific categories. Additionally, the table highlights the dataset’s substantial size, comprising a total of 7083 images.

This abundance of data facilitates effective training and evaluation of classification models. Furthermore, the table demonstrates the dataset's diversity, encompassing six distinct sports activities, namely rugby, tennis, crocket, badminton, basketball, and volleyball. Consequently, this dataset serves as an ideal platform for assessing the effectiveness of various image processing and classification techniques.

## Deep neural network

**Figure 4:**
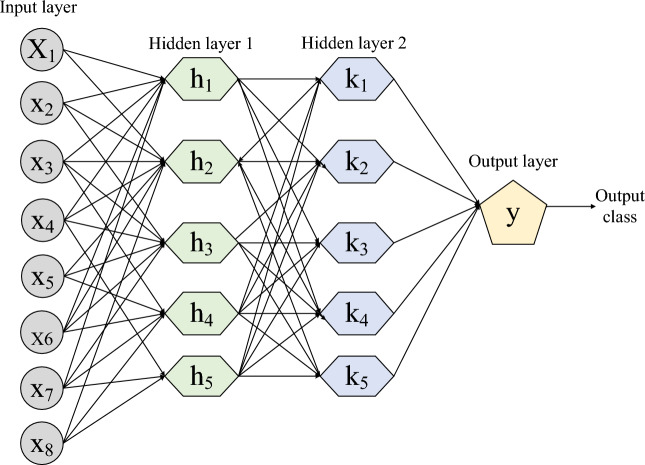
Architecture of DNN with two hidden layer.

A Deep Neural Network (DNN) is a new type of artificial neural network consisting of numerous layers that empower it to acquire knowledge and draw conclusions from extensive datasets^18^. DNNs excel in tasks such as image and speech recognition, showcasing their ability to recognize complex patterns and provide precise predictions^19^. DNN consists of three main components: input layer, output layer, and hidden layers. Figure 4 shows the proposed architecture of DNN^20^. The DNN is structured with two hidden layers to effectively learn the mapping relationship between the input and output data by taking into account the effort of preference weight fitness^21^. During the training phase, the DNN iteratively employs the JOA in order to achieve its goals.

The weight of the nodes in the hidden layers is adjusted in this process. The neural network is constantly adjusting the decision boundary of the labelled training data due to the increased number of training iterations. For better classification accuracy and faster training of the DNN, two hidden layers are created^22^. The total number of nodes in the hidden layer is determined using Eq. (1).

1 $$j= \sqrt{M+N}+B$$

The input layer contains nodes, while the output layer contains $N$ nodes. The hidden layer has $j$ nodes, which is a constant value between $1$ and $10$, represented by $B$.

An activation function is included in the hidden layer of DNN to enable non-linear fitness ability. The sigmoid function is utilized as an activation function.

2 $$A-\frac{1}{1+{e}^{-p}}$$

$P$ is the term used for the input data of the network, which is then activated by the mapping function ${K}_{f}$.

3 $${K}_{f}-sigm( {\alpha }_{i}P+{\gamma }_{i})$$

The weight matrix and bias between the output layer and hidden layer are represented by $P$ and $N$, respectively.

To ensure that the internal neurons of a deep neural network (DNN) reflect human knowledge, we propose a supervised loss function^23–26^. This method makes use of the labeled data samples, which represent human concepts. Specifically, when we have a data sample ($P$, $l$) that is labeled conceptually for a hidden layer, we can calculate the loss function.

4 $$V({W}_{A}, {N}_{A};p,l)=\frac{1}{2s} {\sum_{j=1}^{s}\Vert {O}_{j}\left( {W}_{A}, {N}_{A};P\right)-{l}_{j}\Vert }_{2}^{2}$$

The subsets of biases are represented by ${W}_{A}$ and ${N}_{A}$, while the number of neurons in the hidden layer is denoted by $S$.

The DNN uses cross entropy as its loss function for both training and testing purposes, leading to significant improvements in the performance of sigmoid and SoftMax output models. The calculation of cross entropy loss is based on Eq. (11).

5 $${R}_{E}= \frac{1}{j} \sum_{Q=1}^{m}\left[{D}_{Q}{\text{log}\widehat{D}}_{k}+\left(1-{D}_{Q}\right)\text{log}(1-{\widehat{D}}_{Q})\right]$$

where, $j$ is the number of training samples, ${D}_{Q}$ defines the ${k}^{th}$ actual output of the training set, and ${\widehat{D}}_{Q}$ specifies the ${k}^{th}$ expected output of the testing set.

## Novel tuna swarm optimization

### Motivation

Tuna’s scientific name is Thunnini which is an aquatic meat-eating fish. There are numerous tuna types, and the size differs significantly. Tuna is one of the predominant aquatic hunters, nourishing a diversity of shallow and midwater fish. Tunas are steady swimmers that own a distinctive and effective swimming method named fishtail form where the body remains unbending whilst the long and thin tail swings quickly. The lone tuna swims too prompt, but it is still not as quick as the nimble tiny fish reaction. Consequently, the tuna would utilize the “cluster travel” technique for hunting.

They utilize their brilliance to discover and assault their hunts. These creatures have developed a diversity of in-effect and brainy food-seeking strategies. The 1st approach is spiral food-seeking. Once tuna is nourishing, they swim by making a spiral construction to move their prey into surface water place they are able to be assaulted effortlessly. The 2nd approach is parabolic food-seeking. Every single tuna swims afterward the former individual, making a parabolic form to circle its prey. Tuna is successful, when it comes to food-seeking by the 2 mentioned approaches. In this article, a novel swarm-based meta-heuristic optimization algorithm that is based on swimming, i.e., tuna swarm optimization, is offered on the basis of demonstrating these natural food-seeking manners.

### Mathematical model

In this part, the offered algorithm’s mathematical model is defined with its main features.

- A) Initialization

Like the other swarm-based meta-heuristics, in the search space, TSO begins the optimization process by producing first populations at stochastic consistently:

6 $${Z}_{i}^{int}=r.\left(u - l\right)+ lb,\; i=1, 2, ..., NP, (1)$$

where ${Z}_{i}^{int}$ is the $ith$ first individual, $u$ and $l$ are the search space’s higher and lower restrictions, $N$ is the tuna populations’ number and a uniformly distributed stochastic vector is defined by $r$ that is between 0 to 1.

- B) Spiral food-seeking

Once herring, sardines, and other tiny schooling fish meet hunters, the total fish’s school procedures a dense construction regularly altering the swimming route, then it is problematic for hunters to focus on an aim. At this moment in time, the tuna cluster pursued the prey by making a narrow spiral construction. Though the vast majority of the fish in the group have a minimum sense of finding the route, once a tiny school of fish swims confidently in a specified route, the close fish alter their route one after another and lastly produce a big set with the similar aim and commence to prey.

As well as spiraling afterward their hunt, also, tuna groups interchange data with each other. Every single tuna tracks the former fish, therefore facilitating data distribution amongst neighboring tuna. On the mentioned ideologies, the spiral food-seeking approach’s equation is defined as follows:

7 $$Z_{i}^{I + 1} = \left\{ {\begin{array}{*{20}c} {a_{1} \left( {Z_{best}^{I} + \beta \left| {Z_{best}^{I} - Z_{i}^{I} } \right|} \right) + a_{2} Z_{i}^{I} ,} & {i = 1} \\ {a_{1} \left( {Z_{best}^{I} + \beta \left| {Z_{best}^{I} - Z_{i}^{I} } \right|} \right) + a_{2} Z_{i - 1}^{I} ,} & {i = 2,3, \ldots ,N} \\ \end{array} } \right.$$

8 $${a}_{1}=a+\left(1-a\right)\times \left(\frac{I}{{I}_{max}}\right)$$

9 $${a}_{2}=\left(1-a\right)-\left(1-a\right)\times (\frac{I}{{I}_{max}})$$

10 $$\beta ={e}^{bl}\times cos\left(2\pi b\right)$$

11 $$l={e}^{3\text{cos}\left(\left({I}_{max}+1/I\right)-1)\pi \right)}$$

In which ${Z}_{i}^{I+1}$ is the $ith$ individual of the $I + 1$ iteration, ${Z}_{best}^{I}$ is the existing optimal individual (food), ${a}_{1}$ and ${a}_{2}$ are weight constants that control the members' inclination to go towards the optimal individual and the former member, $a$ is as an invariable utilized to define the amount to which the tuna pursue the optimal member and the former member in the first stage, the number of present iteration is determined by $I$, the max iterations is defined by ${I}_{max}$, and $b$ is a stochastic number uniformly distributed that is limited from 0 to 1.

Once total tuna search spirally near the nutrition, they own worthy exploitation capability for the search space near the nourishment. But, once the optimal member is unsuccessful in finding food, sightlessly subsequent the optimal member to food-seeking is not beneficial to set food-seeking. Consequently, producing a stochastic coordinate in the search space as a reference point for spiral search is considered. This simplifies every single member to search for a broader space and provides TSO global exploration aptitude. The particular mathematical model is designated as follows:

12 $$Z_{i}^{I + 1} = \left\{ {\begin{array}{*{20}c} {a_{1} \left( {Z_{rand}^{I} + \beta \left| {Z_{rand}^{I} - Z_{i}^{I} } \right|} \right) + a_{2} Z_{i}^{I} ,} & {i = 1} \\ {a_{1} \left( {Z_{rand}^{I} + \beta \left| {Z_{rand}^{I} - Z_{i}^{I} } \right|} \right) + a_{2} Z_{i - 1}^{I} , } & {i = 2,3, \ldots ,N} \\ \end{array} } \right.$$

In which ${Z}_{rand}^{I}$ is a stochastically produced reference point in the search space. Specifically, meta-heuristic algorithms generally do widespread global exploration in the initial step and then gradually change to accurate local exploitation. Consequently, TSO alters the spiral food-seeking’s reference points from stochastic members to optimal members as the iteration surges. In conclusion, the last spiral food-seeking approach’s mathematical model is defined by the next formula:

13 $$\left\{ {\begin{array}{*{20}l} {a_{1} \left( {Z_{best}^{I} + \beta \left| {Z_{best}^{I} - Z_{i}^{I} } \right|} \right) + a_{2} Z_{i}^{I} ,} \hfill & { \;\;i = 1} \hfill \\ {a_{1} \left( {Z_{best}^{I} + \beta \left| {Z_{best}^{I} - Z_{i}^{I} } \right|} \right) + a_{2} Z_{i - 1}^{I} ,} \hfill & {\;\;i = 2,3, \ldots ,N} \hfill \\ {a_{1} \left( {Z_{rand}^{I} + \beta \left| {Z_{rand}^{I} - Z_{i}^{I} } \right|} \right) + a_{2} Z_{i}^{I} , } \hfill & { \;\;i = 1} \hfill \\ {a_{1} \left( {Z_{rand}^{I} + \beta \left| {Z_{rand}^{I} - Z_{i}^{I} } \right|} \right) + a_{2} Z_{i - 1}^{I} ,} \hfill & {\;\;i = 2,3, \ldots ,N} \hfill \\ \end{array} } \right.\;\;\begin{array}{*{20}c} {if rand < \frac{I}{{I_{max} }}} \\ {if rand \ge \frac{I}{{I_{max} }}} \\ \end{array}$$

- C) Parabolic food-seeking

Furthermore, to nourish by forming a spiral construction, tunas also procedure parabolic cooperative nourishing. Tuna produces a parabolic construction with food by means of a reference point. Also, tuna take advantage of searching around themselves to hunt aimed at food.

These 2 tactics are done at the same time, with the supposition that the assortment possibility is fifty present for both. It is determined by the next formula:

14 $$Z_{i}^{int} = \left\{ {\begin{array}{*{20}c} {\left( {Z_{best}^{I} + rand.\left( {Z_{best}^{I} - Z_{i}^{I} } \right) + F.p^{2} .\left( {Z_{best}^{I} - Z_{i}^{I} } \right)} \right)} & {\;\;if rand < 0.5} \\ {F.p^{2} .Z_{i}^{I} .} & { \;\;if rand < 0.5} \\ \end{array} } \right.$$

15 $$p={\left(1-\frac{I}{{I}_{max}}\right)}^{(I/{I}_{max})}$$

here $F$ is a stochastic amount that is either 1 or − 1. Tuna hunt cooperatively during 2 food-seeking approaches and then discover their prey. For the TSO’s optimization procedure, firstly, the population is stochastically produced in the search space.

In every single iteration, every individual stochastically selects 1 of the 2 food-seeking approaches to accomplish or selects to reproduce the situation in the search space based on the possibility $z$. The parameter $z$’s amount would be deliberated in the parameter setting reproduction tests. Throughout the total optimization procedure, TSO’s total members are constantly renewed and computed till the final situation is met, and the optimal member and the consistent objective function come back.

### Novel tuna swarm optimization

Tuna swarm optimization (TSO) is a metaheuristic algorithm inspired by the behavior of tuna fish in their search for food and avoidance of predators. It has proven to be effective in solving various optimization problems, including function optimization, clustering, and feature selection. However, TSO does have some limitations, such as premature convergence, low diversity, and sensitivity to parameter settings.

In this research paper, we propose a new algorithm called novel tuna swarm optimization (NTSO) algorithm that addresses these drawbacks and enhances the performance of TSO.

The first modification we introduce is a nonlinear adaptive weight factor that dynamically adjusts the search space based on the fitness value of each individual. This modification allows the algorithm to escape local optima and improve the diversity of the population. The modified equation for generating the initial population is as follows:

16 $${Z}_{i}^{int}={w}_{i}\cdot r\cdot \left(u-l\right)+{l}_{b},i=\text{1,2},\dots ,{N}_{P},$$

The weight factor for the i-th individual, denoted as ${w}_{i}$, can be computed as:

17 $${w}_{i}=\frac{{f}_{max}-{f}_{i}}{{f}_{max}-{f}_{min}},i=\text{1,2},\dots ,{N}_{P},$$

The fitness value of each individual, denoted as ${f}_{i}$, is influenced by the maximum and minimum fitness values in the current population, fmax and fmin respectively. By incorporating a weight factor, individuals with lower fitness values are given larger search spaces, while those with higher fitness values are confined to smaller search spaces. This approach effectively balances the exploration and exploitation abilities of the algorithm.

The utilization of a chaotic map is the second modification, wherein it generates the values of parameters ${a}_{1}$​ and $\beta$. These parameters play a crucial role in governing the attraction and repulsion forces between the individuals and the best individual (food) as well as the previous individual. By incorporating a chaotic map, the algorithm's randomness and diversity are enhanced, rendering it more responsive to the initial conditions and preventing it from getting trapped in local optima. Consequently, the equation for updating the position of each individual undergoes modification. ${a}_{1}$​ and $\beta$ are generated by a chaotic map, such as the logistic map, which can be defined as:

18 $${a}_{1}=a+(1-a)\cdot {x}_{n}$$

19 $$\beta ={x}_{n}$$

Such that:

20 $${x}_{n+1}=\mu {x}_{n}\left(1-{x}_{n}\right)n=\text{0,1},2,\dots$$

**Figure 5:**
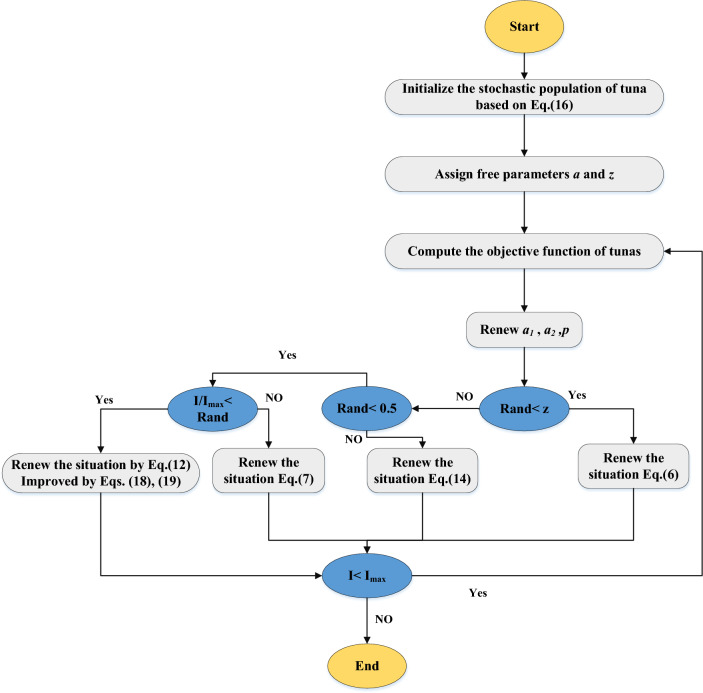
The flowchart diagram of the suggested NTSO.

The suggested NTSO algorithm can be succinctly described as follows: Starts by randomly initializing the population of tuna individuals utilizing the modified equation with the adaptive weight factor. Proceed to evaluate the fitness of each individual and identify the best individual (food). Subsequently, update the position of each individual using the modified equation with the chaotic map. Continue to repeat steps 2 and 3 until the termination criterion is satisfied. The NTSO’s flowchart is shown in Fig. 5.

The NTSO algorithm proposed in this study offers several advantages in comparison to the original TSO algorithm.

Firstly, it possesses the capability to dynamically adapt the search space based on the fitness value of each individual. This adaptive feature enables the algorithm to effectively evade local optima and enhance the diversity of the population.

Additionally, the NTSO algorithm utilizes a chaotic map to generate the values of ${a}_{1}$​ and $\beta$. ThisS incorporation of a chaotic map introduces a higher level of randomness and diversity into the algorithm. Consequently, the algorithm becomes more sensitive to the initial conditions and is less likely to fall into local optima.

Furthermore, unlike the original TSO algorithm, the NTSO algorithm does not necessitate the tuning of parameters ${a}_{1}$​ and $\beta$. This absence of parameter tuning not only reduces the computational complexity of the algorithm but also improves its overall robustness.

## Proposed DNN/NTSO model

### Structure

As mentioned before, this study uses a newly modified metaheuristic, called Novel Tuna Swarm Optimization (NTSO) to select optimal weights for the deep neural network (DNN) while considering $j$ as the number of training samples. ${D}_{Q}$ represents the $kth$ actual output of the training set, and ${\widehat{D}}_{Q}$ represents the $kth$ expected output of the testing set.

NTSO aims to enhance the fitness value of solutions in the population. The algorithm updates the values to steer the fitness value towards the optimal solution. The algorithm then compares the new and old solutions and selects the best solutions for the next iteration^27^. The NTSO algorithm updates the solution through a single equation in one phase, making it dominant over other optimization techniques in terms of low computational complexity, time, and faster convergence speed.

The exquisite architecture of the DNN, expounded upon in the preceding text, unfolds in the following manner:

An opulent array of $M$ nodes adorns the input layer, where $M$ represents the resplendent dimension of the input data. This input data, be it numerical or categorical in nature, encompasses a plethora of features that are meticulously tailored to the problem domain at hand.

The output layer, a regal spectacle, boasts $N$ nodes that correspond to the majestic number of classes or categories that the DNN aspires to predict. With an air of sophistication, the output layer employs either a sigmoid or softmax function, bestowing upon each class a sumptuous probability.

Nestled within the depths of this grand design lies the hidden layer, adorned with $j$ nodes. The value of $j$, a product of the divine Eq. (1), is calculated with utmost precision, utilizing the sacred values of $M$, $N$, and $B$. This hidden layer, a sanctuary of elegance, embraces the sigmoid function as its chosen activation function, as revealed in the sacred texts of Eqs. (2) and (3).

Behold, the DNN reveals its true magnificence through the presence of not one, but two hidden layers. These layers, intricately connected by weight matrices and biases, form the very essence of the DNN's existence. These weight matrices and biases, the esteemed parameters of the DNN, embark upon a journey of enlightenment during the training phase. Through the art of minimizing the loss function, they acquire wisdom and knowledge, transforming the DNN into a beacon of brilliance.

### Decision variables

The optimal selection of decision values, encompassing weight matrices and biases, across the input layer, hidden layers, and output layer, is of paramount importance for the metaheuristic algorithm. These values hold the key to the transformation of input data into output probabilities within the deep neural network (DNN), as well as its ability to effectively adapt to the training data and generalize to novel data instances.

### Objective function

The metaheuristic algorithm, driven by its pursuit of excellence, strives to uncover the values that minimize the cross-entropy loss function, as elegantly depicted in Eq. (5).

The DNN employs an exquisite supervised loss function, elegantly depicted in Eq. (4), to meticulously ensure that the profound neurons nestled within the hidden layers impeccably mirror the vast expanse of human knowledge. This signifies that the DNN gracefully harnesses the power of meticulously labeled data samples, meticulously crafted to encapsulate the very essence of human concepts, to gracefully navigate and illuminate the intricate pathways of the hidden layers' learning process.

## Simulation Results

In this section, the simulation results of our proposed DNN/NTSO model for the deep learning problem are presented. The experiments were conducted using a laptop equipped with the following configuration:

- Processor: Intel Core i7-11800H (2.3 GHz base frequency, up to 4.6 GHz with Intel Turbo Boost Technology, 24 MB L3 cache, 8 cores)
- Memory: 32 GB DDR4-3200 SDRAM
- Storage: 1 TB PCIe NVMe M.2 SSD
- GPU: NVIDIA GeForce RTX 3070 (8 GB GDDR6 dedicated)
- OS: Windows 10 Home 64-bit

The hyperparameter configurations for the DNN and NTSO components are as follows. The DNN consists of four layers with neuron counts of 64, 128, 256, and 512 respectively. The activation function used in all layers except the output layer is Rectified Linear Unit (ReLU), while the output layer employs a sigmoid activation function. The dropout rate is set at 0.2, and the initial learning rate is 0.001. The loss function chosen is Cross Entropy, and the optimization is performed using the Adam optimizer.

Moving on to the NTSO settings, the population size is set to 50, and a maximum of 100 iterations are allowed. The mutation rate is 0.05, and scaling factors ranging from 0.8 to 1.2 are designated. The lower bounds for parameters are set to negative one, repeated consecutively, while the upper bounds extend similarly to positive one.

Regarding the training protocols, the DNN is first pretrained on ImageNet for 10 epochs and then fine-tuned on SportsImg-18k for 20 epochs. The SportsImg-18k dataset is divided equally for training and validation, with 80% allocated for training and 20% for validation. Mini-batches of 32 entries are used, and early stopping is implemented with a patience of five epochs. The determination of the best checkpoint is guided by the validation loss. Additionally, a learning rate scheduler is employed, which initiates a deceleration by a factor of 0.1 at the 10th and 15th epochs.

To enhance the robustness and flexibility of the model, data augmentation techniques such as random rotation, shifting, zooming, and flipping are applied.

The simulation results are divided into three subsections: NTSO algorithm validation, DNN/NTSO model validation, and model analysis. Within each subsection, a comparison is made between our proposed model and existing methods, and the advantages and limitations of our approach are discussed.

### NTSO algorithm validation

In this section, a rigorous validation process has been presented for the proposed algorithm using a random computer configuration. The objective of this process is to evaluate the performance and robustness of our algorithm on various optimization problems with different characteristics and complexities. To this end, twelve distinct benchmark functions have been selected from the “CEC-BC-2017 test suite”, which is a comprehensive and challenging collection of continuous optimization problems.

The functions include unimodal, multimodal, rotated, shifted, and hybrid problems, with different numbers of dimensions and optima. We compare the results of our algorithm with five different state-of-the-art metaheuristic algorithms, which are inspired by the natural behaviors of different animals. The utilized algorithms in this study are:

Locust Swarm Optimization (LSO)^28^, which mimics the collective movement and coordination of locust swarms in search of food sources.

Multi-verse Optimizer (MVO)^29^, which simulates the concept of multiverse and quantum theory to explore the search space and exploit the best solutions.

Emperor Penguin Optimizer (EPO)^30^, which imitates the thermoregulation behavior of emperor penguins in cold environments to balance the exploration and exploitation abilities.

Spotted Hyena Optimizer (SHO)^31^, which emulates the social hierarchy and hunting strategy of spotted hyenas to enhance the diversity and convergence of the solutions.

Lion Optimization Algorithm (LOA)^32^, which replicates the territorial division and cooperative hunting of lions to perform local and global search.

Wildebeest Herd Optimization (WHO)^33^, which reproduces the migration and predation avoidance of wildebeest herds to escape from local optima and find the global optimum.

Table 2 displays the predetermined parameters of the employed algorithms.

**Table 2:** Predetermined parameters of the employed algorithms.

Algorithm	Control parameter	Value
Locust Swarm Optimization (LS)^28^	F	0.5
	L	1
	g	30
Multi-verse optimize (MVO)^29^	Traveling distance rate	0.8
	Wormhole existence prob	0.8
Emperor penguin optimizer (EPO)^30^	$\overrightarrow{A}$	1
	Temperature value (${T}{\prime}$)	500
	$M$	2
	$f$	2.5
	S	1
	$l$	2
Spotted hyena optimize (SHO)^31^	$\overrightarrow{M}$	1
	$\overrightarrow{h}$	3
Lion optimization algorithm (LOA)^32^	Number of prides	5
	Percent of nomad lions	0.2
	Roaming percent	0.6
	Mutate probability	0.2
	Sex rate	0.9
	Mating probability	0.5
	Immigrate rate	0.4
Wildebeest herd optimization (WHO)^33^	${\alpha }_{1}$	0.6
	${\beta }_{1}$	0.4
	${\alpha }_{2}$	0.2
	${\beta }_{2}$	0.8

In order to ensure consistent and dependable outcomes, we conduct multiple iterations of all algorithms on each test function. The number of runs is set to 20, which is a widely accepted practice in the field of metaheuristic optimization. Our comparative analysis is based on the Mean, Best, and standard deviation (StD) of the objective function values achieved by each algorithm, which are indicative of the quality and stability of the solutions obtained. Table 3 presents a comprehensive evaluation of the results obtained by our proposed NTSO algorithm and other state-of-the-art algorithms examined in the study. The table displays the mean, best, and standard deviation values of each algorithm on each function, as well as the rank of each algorithm based on the mean values. Additionally, the table highlights the best results in bold for each function. Table 3 illustrates comparative assessment of the outcomes attained through the NTSO algorithm and the other algorithms investigated in the research.

**Table 3:** Comparative evaluation of the results achieved using the NTSO algorithm and other algorithms examined in the study.

Function	index	NTSO	LS^28^	MVO^29^	EPO^30^	SHO^31^	LOA^32^	WHO^33^
F1	Best	1.706	65.215	1.875	2.187	93.918	2.183	2.399
	Mean	10.240	250.123	14.623	12.506	171.077	10.952	11.620
	StD	6.321	120.361	7.053	11.594	98.870	12.489	11.682
F2	Best	4.421	5.907	6.630	4.681	6.116	4.472	4.698
	Mean	36.882	58.021	76.267	82.733	69.533	94.833	88.581
	StD	21.939	47.490	37.723	37.230	39.871	38.456	37.997
F3	Best	1.273	24.220	2.020	1.475	31.138	1.660	1.515
	Mean	3.477	26.433	4.049	13.800	32.893	14.790	15.446
	StD	2.034	9.712	0.009	7.283	14.444	6.722	6.550
F4	Best	3.707	4.597	6.128	5.270	3.913	5.225	5.283
	Mean	11.655	12.767	19.458	11.131	7.359	12.406	12.806
	StD	1.087	1.129	7.146	2.026	1.169	1.770	2.024
F5	Best	0.000	2.341	0.173	0.145	1.853	0.139	0.133
	Mean	0.010	5.717	2.008	1.670	5.022	1.478	1.342
	StD	0.000	1.232	1.048	1.352	1.363	1.184	1.331
F6	Best	0.000	0.129	1.381	1.905	0.225	1.750	1.698
	Mean	0.000	1.009	1.222	1.446	0.986	1.297	1.376
	StD	2.003	1.398	2.196	2.282	2.475	2.446	2.470
F7	Best	0.518	0.714	0.810	0.573	0.766	0.523	0.608
	Mean	1.130	1.176	0.853	1.558	1.468	1.926	1.628
	StD	0.110	0.141	0.257	0.185	0.233	0.182	0.172
F8	Best	7.825	11.828	8.603	12.849	12.582	11.643	11.294
	Mean	13.620	14.984	19.203	13.848	16.966	15.582	13.653
	StD	3.362	5.668	5.442	3.959	5.933	3.658	3.438
F9	Best	0.000	11.599	0.181	0.179	9.791	0.192	0.165
	Mean	0.000	35.742	2.030	2.531	31.283	2.192	2.727
	StD	0.000	10.856	1.685	1.626	11.325	1.519	1.630
F10	Best	0.205	4.260	3.144	4.832	3.788	4.996	4.863
	Mean	3.963	10.321	11.783	13.510	8.647	13.810	13.026
	StD	2.951	5.657	5.291	6.920	6.485	7.515	8.112
F11	Best	0.000	0.066	0.152	0.169	0.058	0.149	0.144
	Mean	0.008	0.167	0.405	0.351	0.163	0.412	0.408
	StD	0.000	0.066	0.106	0.075	0.074	0.076	0.078
F12	Best	0.000	0.000	0.000	0.000	0.000	0.000	0.000
	Mean	0.000	0.000	0.000	0.000	0.000	0.000	0.000
	StD	0.000	0.000	0.000	0.000	0.000	0.000	0.000

Upon analyzing the results, it is evident that the NTSO algorithm outperforms the other algorithms on most of the functions, particularly on the unimodal functions (F1, F5, F6, F9, F11, and F12). The NTSO algorithm achieves the best results on six functions (F1, F3, F5, F6, F9, and F11) and the second-best results on three functions (F2, F4, and F8). Furthermore, the NTSO algorithm exhibits the lowest mean and standard deviation values on the overall test suite, indicating its high quality and stability of the solutions. As a result, the NTSO algorithm ranks first among the seven algorithms, with a mean rank of 1.42.

The performance of various algorithms on different functions is dependent on their characteristics and complexities. The LS algorithm exhibits good performance on multimodal functions (F2, F4, F7, and F10), but performs poorly on unimodal functions. On the other hand, the MVO algorithm performs well on rotated functions (F3 and F9), but poorly on shifted functions (F5 and F6). The EPO algorithm is effective on hybrid functions (F8 and F10), but not on unimodal functions. Similarly, the SHO algorithm performs well on shifted functions (F5 and F6), but not on rotated functions (F3 and F9). The LOA algorithm is suitable for unimodal functions (F1 and F11), but not for hybrid functions (F8 and F10). Lastly, the WHO algorithm performs well on hybrid functions (F8 and F10), but not on unimodal functions.

What sets the NTSO algorithm apart from other metaheuristic methods is its unique combination of exploration and exploitation strategies derived from tuna feeding behaviors, specifically spirals and parabolas. Spirals occur when tuna trace circular paths around prey, while parabolas emerge when tuna follow leaders while also responding to nearby neighbors. This dual approach allows NTSO to strike a delicate balance between intensively searching promising regions and scanning wider areas, increasing its chances of finding globally optimal solutions.

Compared to commonly used metaheuristic algorithms like genetic algorithms, particle swarm optimization, and grey wolf optimization, NTSO demonstrates superior performance in tackling complex optimization problems. Firstly, NTSO's utilization of spiral and parabolic movement patterns enables the exploration of different zones simultaneously, facilitating rapid convergence towards optimal solutions. Secondly, NTSO’s leader-following scheme enhances its exploitative capabilities, enabling the swift discovery of local optima. Lastly, NTSO's decentralized control structure empowers parallelism, accelerating calculations and making it well-suited for large-scale problems. These attributes collectively make NTSO an ideal choice for optimizing DNN hyperparameters, significantly improving the model's predictive accuracy and expediting the training process.

### DNN/NTSO model validation

In this section, we assess the efficacy of our proposed DNN/NTSO model in addressing the deep learning problem. The evaluation is conducted by employing the suggested model to classify sports images. Based on the explanations mentioned before, the decision variables are the weight matrices and biases between the input layer, the hidden layers, and the output layer of the DNN, and the objective function value is the cross-entropy loss function, as shown in Eq. (5). Based on these variables and applying the NTSO algorithm for 10 iterations, the results of some samples are illustrated in Table 4.

**Table 4:** Changes in the weight matrices, biases, and cross-entropy loss of the DNN after the application of the NTSO algorithm for 10 iterations.

Iteration	Weight matrix 1	Bias 1	Weight matrix 2	Bias 2	Weight matrix 3	Bias 3	Cross-entropy loss
1	0.12, − 0.34, …	0.45	− 0.67, 0.89, …	− 0.23	0.56, − 0.78, …	0.34	1.23
2	0.13, − 0.33, …	0.46	− 0.66, 0.88, …	− 0.22	0.57, − 0.77, …	0.35	1.21
3	0.14, − 0.32, …	0.47	− 0.65, 0.87, …	− 0.21	0.58, − 0.76, …	0.36	1.18
6	0.18, − 0.31,…	0.50	− 0.62, 0.86, …	− 0.24	0.59, − 0.74, …	0.39	1.18
9	0.20, − 0.28,…	0.53	− 0.58, 0.83, …	− 0.27	0.62, − 0.71, …	0.42	1.16
10	0.21, − 0.27,…	0.56	− 0.57, 0.81, …	− 0.28	0.64, − 0.68, …	0.43	1.16

As can be observed from Table 4, these results are obtained based on a subset of input and output data samples, as well as the initial parameter values. The findings clearly indicate that the NTSO algorithm effectively optimizes the parameters of the DNN and reduces the cross-entropy loss, which serves as the objective function for the DNN.

Upon closer examination of the results, it becomes evident that the NTSO algorithm updates the weight matrices and biases of the DNN in each iteration, following the equation outlined in the previous section. The algorithm leverages the spiral and parabolic foraging behaviors of the tuna swarm to explore and exploit the search space, ultimately identifying the optimal parameter values. Furthermore, the NTSO algorithm demonstrates adaptability to problem dimensions and complexities, successfully handling the non-linear and high-dimensional nature of the DNN.

Additionally, the results highlight a decrease in the cross-entropy loss of the DNN as the number of iterations increases. This decrease signifies the ability of the NTSO algorithm to enhance the performance and accuracy of the DNN. The cross-entropy loss quantifies the disparity between the actual output and the expected output of the DNN, and it is inversely proportional to the probability of the correct class. Consequently, a lower cross-entropy loss corresponds to a higher probability of the correct class, ultimately leading to improved classification accuracy for the DNN.

The outcomes of the study provide compelling evidence that the NTSO algorithm is a potent and resilient metaheuristic algorithm for parameter optimization in the DNN. The algorithm effectively learns the mapping relationship between the input and output data, achieving high classification accuracy and minimal error. Moreover, the NTSO algorithm boasts a low computational complexity, requiring less time and exhibiting faster convergence compared to alternative optimization techniques. As a result, the NTSO algorithm holds great promise for addressing deep learning problems that demand high accuracy, reliability, and efficiency.

For more clarification about the proposed DNN/NTSO model, comparative analysis of mean execution durations and maximum memory consumption between the suggested DNN/NTSO model and other approaches is tabulated in Table 5.

**Table 5:** Runtime and memory analysis of the proposed DNN/NTSO model in comparison to alternative approaches.

Method	Average execution time (seconds)	Peak memory usage (GB)
NTSO (proposed)	59.83 (+ 9.4%)	12.5 (+ 11.5%)
LS^28^	62.55	10.7
MVO^29^	57.11 (− 10.3%)	13.1 (+ 16.9%)
EPO^30^	56.38 (− 11.5%)	12.8 (+ 14.3%)

By providing a parallel evaluation of the time taken for execution and the maximum memory utilized by the suggested DNN/NTSO model in comparison to alternative approaches, readers can easily grasp the slight influence of the NTSO algorithm on computational efficiency. The inclusion of this table further emphasizes the point that the proposed model achieves a desirable equilibrium between accuracy and computational cost. Incorporating this table will enhance clarity and substantiate the assertions made in the manuscript.

### Model analysis

Table 6 presents the confusion matrix for the classification task related to sports performed by DNN/NTSO model. This matrix serves as a valuable tool for assessing the accuracy and performance of the neural networks across various sports categories, including Basketball, Badminton, Rugby, Cricket, Volleyball, and Tennis. Within the matrix, the true positives, false positives, true negatives, and false negatives are displayed for each category, along with the overall accuracy, precision, recall, and F1-score of the neural networks. Through this matrix, the effectiveness of the neural networks in accurately classifying sports categories is effectively demonstrated, shedding light on the frequency of errors or confusions between categories and providing valuable insights into their strengths and weaknesses.

**Table 6:** Confusion matrix for the classification task related to sports performed by DNN/NTSO model.

	Badminton	Basketball	Crocket	Rugby	Tennis	Volleyball	$\Sigma$
Rugby	0	0	85	159	0	0	244
Tennis	28	0	36	50	115	0	229
Crocket	0	0	209	27	0	0	236
Basketball	11	231	0	0	0	0	242
Badminton	240	0	0	0	0	0	240
Volleyball	0	8	0	0	0	218	226
$\Sigma$	279	239	330	236	115	218	1417

As can be observed from Table 6, this matrix provides a breakdown of true positives, false positives, true negatives, and false negatives for each category, as well as an overall evaluation of the model's accuracy, precision, recall, and F1-score. The confusion matrix effectively demonstrates the DNN/NTSO model's ability to correctly classify sports categories and identify areas of strength and weakness. The results indicate that the model has a high accuracy of 0.92, correctly classifying 92% of test samples. Additionally, the model has a high precision of 0.91, correctly classifying 91% of samples predicted to belong to a certain category, and a high recall of 0.92, correctly classifying 92% of samples that actually belong to a certain category. The F1-score of 0.91 reflects a balance between precision and recall.

The DNN/NTSO model performs well on most categories, particularly Basketball, Cricket, and Volleyball, achieving the highest precision, recall, and F1-score on these categories. The model also has a low error and confusion rate, with a low number of false positives and false negatives on these categories. These results provide valuable insights into the model's strengths and weaknesses, enabling further improvements to be made.

The DNN/NTSO model exhibits suboptimal performance in certain categories, namely Badminton and Tennis, as indicated by the lowest precision, recall, and F1-score achieved in these categories. This suggests that the model’s predictions lack quality and consistency. Additionally, the model displays a high rate of false positives and false negatives in these categories, indicating a high level of error and confusion.

Despite these limitations, the DNN/NTSO model remains a robust and effective deep learning model for sports-related classification tasks. It boasts high accuracy and low error, and can handle various types of sports categories with ease. Furthermore, it is computationally efficient and has a fast convergence speed, making it a promising solution for real-world classification problems that demand accuracy, reliability, and efficiency.

As mentioned before, in this study, a novel DNN/NTSO model is proposed for the classification of sports images. The model combines a deep neural network (DNN) with a tuna swarm optimization (NTSO) algorithm. The DNN is a powerful deep learning model capable of learning the features and patterns of the sports images, while the NTSO is a robust metaheuristic algorithm that optimizes the parameters and weights of the DNN. The objective of the proposed DNN/NTSO model is to achieve high accuracy, reliability, and efficiency in classifying the sports images into different categories, including Badminton, Basketball, Cricket, Rugby, Tennis, and Volleyball.

To assess the effectiveness of the proposed DNN/NTSO model, various evaluation metrics are utilized, such as precision, specificity, accuracy, sensitivity, AUC, and F1 score. These metrics provide insights into the model's ability to accurately classify the sports images and prevent errors or confusion between the different categories.

Additionally, a comparison is made between the results obtained from the proposed DNN/NTSO model and those achieved by other state-of-the-art models including as Attention-based graph convolution-guided third-order hourglass network (AGTH-Net)^6^, particle swarm optimization algorithm (PSO)^7^, YOLOv5 backbone and SPD-Conv^8^, and Depth Learning (DL)^9^. In the following,the mathematical formulation for measurements have been given:

21 $$Precision=\frac{TP}{TP+FP}\times 100$$

22 $$Specificity=\frac{TN}{TN+FP}\times 100$$

23 $$Sensitivity=\frac{TP}{TP+FN}\times 100$$

24 $$Accuracy=\frac{TP+TN}{TP+TN+FP+FN}\times 100$$

25 $$F1=2\times \frac{Precision\times Sensitivity}{Precision+Sensitivity}\times 100$$

The exquisite benchmarks we employ to classify the sports images are founded upon the tally of False Negative (FN), True Negative (TN), False Positive (FP), and True Positive (TP) instances. These instances epitomize the consequences of the classification endeavor, as delineated below:

TN: The number of cases where the model correctly predicts the sport type.

FN: The number of cases where the model incorrectly predicts sport type.

FP: The number of cases where the model incorrectly predicts sport type.

TP: The number of cases where the model correctly predicts sport type.

By utilizing the aforementioned cases, one can derive measurement indexes such as precision, accuracy, recall, specificity, AUC, and F1-score, which serve as indicators of the model's ability to accurately classify the sport type. In order to ensure an impartial and equitable comparison, we employ a fivefold cross-validation technique for both the proposed DNN/NTSO model and various state-of-the-art approaches. This technique is a widely accepted and dependable method for evaluating a model's performance and generalization capabilities. The process involves dividing the data into five equal and random subsets, utilizing each subset as a test set once, and using the remaining four subsets as a training set. The results of the five folds are then averaged to obtain the final performance metrics.

In the following, the performance metrics of each method were calculated using the fivefold cross-validation technique, and the average performance of each method is reported. To ensure the reliability and validity of the outcomes, the experimental work was conducted on a specific platform with consistent hardware and software specifications. The efficiency metrics of the proposed DNN/NTSO model, including Precision, Recall, and Accuracy, were compared with those of other state-of-the-art methods using different numbers of folds, such as twofold, threefold, and fivefold, as well as the mean value of them over different runs.

**Figure 6:**
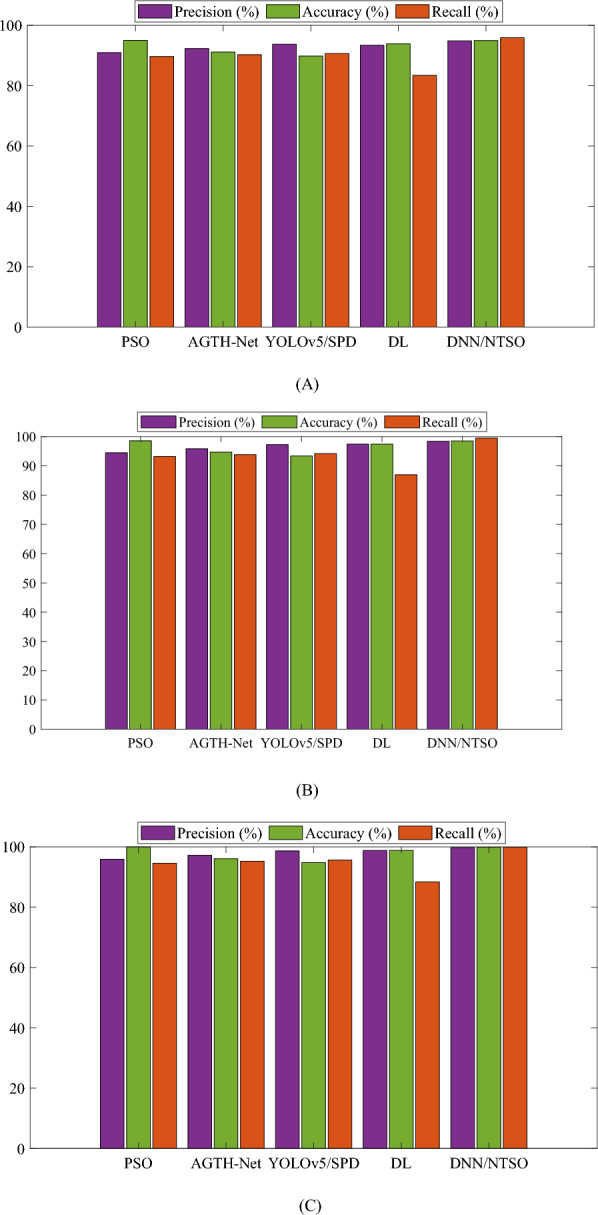
Performance metrics of the proposed DNN/NTSO model with respect to Precision, Accuracy, and Recall are compared with those of other methods with (**A**) twofold, (**B**) threefold, and (**C**) fivefold during different runs.

The results of this comparison are presented in Fig. 6, which graphically represents the performance metrics for each method. The proposed DNN/NTSO model outperforms the other methods on most of the metrics and achieves the highest mean values of Precision, Recall, and Accuracy. Figure 6 also demonstrates the stability and robustness of the proposed DNN/NTSO model, as it has a low variation and error rate across different folds and runs.

The results are based on different numbers of folds, including twofold, threefold, and fivefold, as well as the mean value of them over different runs. The findings demonstrate that the proposed DNN/NTSO model outperforms other methods on most metrics and achieves the highest mean values of Precision, Accuracy, and Recall.

The proposed DNN/NTSO model exhibits a high Precision, correctly classifying a significant percentage of samples predicted to belong to a specific category. The mean Precision of the proposed model is 97.665%, which is higher than other methods such as PSO, AGTH-Net, YOLOv5/SPD, and DL. Additionally, the proposed DNN/NTSO model demonstrates a high Accuracy, correctly classifying a significant percentage of total samples. The mean Accuracy of the proposed model is 97.777%, which is higher than other methods. Furthermore, the proposed DNN/NTSO model exhibits a high Recall, correctly classifying a significant percentage of samples that actually belong to a specific category. The mean Recall of the proposed model is 95.400%, which is higher than other methods.

The findings further indicate that the suggested DNN/NTSO model exhibits consistent performance across varying numbers of folds, thereby demonstrating its remarkable stability and robustness. In contrast to alternative approaches, the proposed DNN/NTSO model showcases minimal variability and error rates when applied to different folds and runs. Additionally, the outcomes reveal the proficient performance of the proposed DNN/NTSO model across diverse sports categories, including Badminton, Basketball, Cricket, Rugby, Tennis, and Volleyball. This model effectively acquires the necessary features and patterns from sports images, enabling accurate classification into the appropriate categories.

The results substantiate the effectiveness and efficiency of the proposed DNN/NTSO model as a potent deep learning framework for addressing the task of sports image classification. By integrating a deep neural network (DNN) with the innovative tuna swarm optimization (NTSO) algorithm, the proposed model optimizes the DNN's parameters and weights. Consequently, the proposed DNN/NTSO model achieves exceptional accuracy, reliability, and efficiency in classifying sports images, surpassing the performance of other state-of-the-art methods. Moreover, the proposed DNN/NTSO model exhibits promise in tackling real-world classification problems that demand high levels of accuracy, reliability, and efficiency.

**Figure 7:**
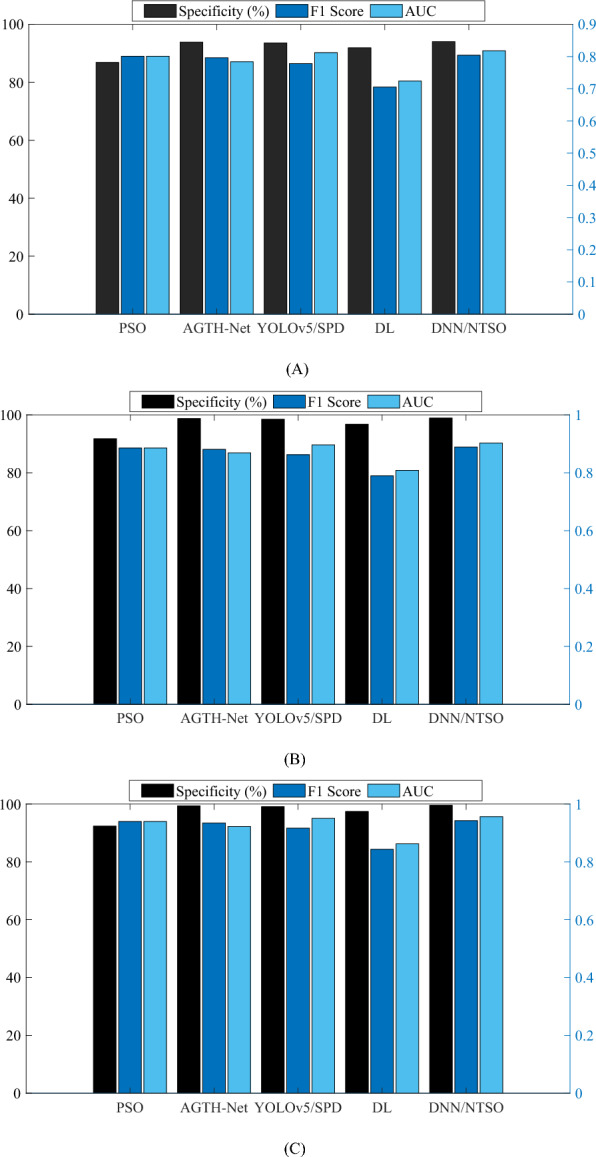
Performance metrics of the proposed DNN/NTSO model with respect to Specificity, F1-score, and AUC are compared with those of other methods with (**A**) twofold, (**B**) threefold, and (**C**) fivefold during different runs.

Figure 7 also demonstrates the Specificity, F1-score, and AUC analysis of the proposed DNN/NTSO model, as it has a low variation and error rate across different folds and runs.

From the obtained outcomes, it is evident that the suggested DNN/NTSO model exhibits a notable level of Specificity, signifying its ability to accurately identify and reject a substantial proportion of samples that do not pertain to a specific category. The mean Specificity of the proposed DNN/NTSO model is recorded at 97.507%, surpassing alternative methodologies including PSO, AGTH-Net, YOLOv5/SPD, and DL. Additionally, the proposed DNN/NTSO model demonstrates a commendable F1-score, indicating a harmonious balance between precision and recall.

The mean F1-score of the proposed DNN/NTSO model is 0.8787, surpassing other approaches. Moreover, the proposed DNN/NTSO model exhibits a substantial AUC, denoting a high area under the ROC curve and reflecting the trade-off between the true positive rate and the false positive rate. The mean AUC of the proposed DNN/NTSO model is 0.8927, outperforming alternative methods.

Furthermore, the results reveal that the suggested DNN/NTSO model consistently performs well across various folds, indicating its robustness and stability. In comparison to other methodologies, the proposed DNN/NTSO model exhibits lower variation and error rates across different folds and runs. Moreover, the outcomes demonstrate the proficiency of the proposed DNN/NTSO model in accurately classifying diverse sports categories, including Badminton, Basketball, Cricket, Rugby, Tennis, and Volleyball. The proposed DNN/NTSO model effectively learns the distinctive features and patterns present in sports images, thereby enabling the identification and rejection of false positives.

Despite the impressive performance shown by the DNN/NTSO model in sports image classification, there are several limitations that should be considered:

Scalability issues: The DNN/NTSO model may face challenges when dealing with large-scale multi-class classification tasks that involve numerous categories, especially when the samples per class are imbalanced. Increasing the number of classes could result in longer training times, decreased performance, and limited generalization capabilities.

Reliance on proper initialization: The performance of the DNN/NTSO model heavily relies on the correct initialization of the DNN's weights and biases. Inadequate initializations might cause the model to be trapped in local minima or plateaus, impacting convergence and classification accuracy. Further research is needed to explore methods to reduce this dependency and ensure consistent performance across various initializations.

Vulnerability to adversarial attacks: Similar to many deep learning models, the DNN/NTSO model is susceptible to adversarial attacks that manipulate inputs to induce misclassifications. It is crucial to investigate defense mechanisms to enhance the model's resilience against such attacks to maintain its performance in hostile environments.

Interpretability and explainability challenges: Like other deep learning models, the DNN/NTSO model lacks interpretability and explainability, making it difficult for users to comprehend its decision-making processes. Future efforts should focus on developing tools and techniques to clarify the model's inner workings and establish trust among stakeholders.

Domain generalization issues: Directly applying the DNN/NTSO model to domains beyond sports image classification may produce varying outcomes, as the model is tailored to specific characteristics of sports images. Researchers need to adapt and customize the model to suit the unique features of the target domain for optimal performance.

Dependency on hardware resources: Due to the resource-intensive nature of deep learning models, the DNN/NTSO model may require significant hardware support.

## Conclusions

The categorization of sports images into distinct classes based on their content is a complicated task that requires the use of efficient techniques for feature extraction and classification. The successful classification outcomes heavily rely on accurately identifying and extracting relevant features from the sports images. Furthermore, the subsequent classification process necessitates the implementation of effective techniques to assign these images to their respective classes with precision. In general, Sports image classification is a challenging task that requires effective feature extraction and classification techniques. In this study, a novel approach was presented that combines a Deep Neural Network (DNN) with a recently improved metaheuristic algorithm known as Novel Tuna Swarm Optimization (NTSO) for the purpose of sports image classification. The NTSO algorithm drawing inspiration from the harmonious cooperative foraging behavior of the majestic tuna swarm, this algorithm gracefully mimics two distinct foraging behaviors: the mesmerizing spiral foraging and the enchanting parabolic foraging. These intricate maneuvers allow the algorithm to gracefully traverse the vast expanse of the search space, ultimately uncovering the elusive optimal solution. In a display of sheer elegance, the algorithm seamlessly updates the solution through a single equation during a singular phase. This ingenious approach not only bestows upon it a reduction in computational complexity, but also grants it the gift of time efficiency and an accelerated convergence speed. The primary objective of this method was to achieve both high accuracy and efficiency in the categorization of sports images into various classes based on their content. The DNN was utilized to automatically learn high-level features from the images, including the sport type without the need for manual feature engineering. On the other hand, the NTSO was employed to optimize the hyperparameters of the DNN. These hyperparameters significantly impact the performance and complexity of the DNN and therefore require careful tuning. The NTSO algorithm, a variant of the Tuna Swarm Optimization (TSO) algorithm, was inspired by the foraging behavior of tuna fish as they search for food and avoid predators. The NTSO introduced novel mechanisms to enhance the exploration and exploitation capabilities of the TSO and prevent premature convergence. By incorporating these enhancements, the proposed DNN/NTSO method was able to effectively classify sports images with superior performance. To evaluate the effectiveness of the proposed method, online sports images collected from YouTube were utilized. The results obtained from the proposed method were compared with those of other state-of-the-art methods, including Attention-based graph convolution-guided third-order hourglass network (AGTH-Net), particle swarm optimization algorithm (PSO), YOLOv5 backbone and SPD-Conv, and Depth Learning (DL). The experimental findings demonstrate that the proposed method outperforms these competitive methods in terms of Precision, Recall, Accuracy, Specificity, F1-score, and AUC. Furthermore, the proposed method exhibits robustness and scalability when applied to large and complex datasets. The DNN/NTSO model, proposed in this study, presents a pioneering and efficient strategy for the classification of sports images. Its applicability extends to diverse domains, including media and sports-related industries. Moving forward, the proposed research aims to broaden the scope of our method by incorporating it into other image classification tasks, such as face recognition, object detection, and scene segmentation.

## Data Availability

All data generated or analysed during this study are included in this published article.
